# Motivation and Problem Solving Versus Mobile 360° Videos to Promote Enrollment in the National Diabetes Prevention Program’s Lifestyle Change Program Among People With Prediabetes: Protocol for a Randomized Trial

**DOI:** 10.2196/28884

**Published:** 2021-06-14

**Authors:** Bryan Gibson, Sara Simonsen, Jonathan Barton, Yue Zhang, Roger Altizer, Kelly Lundberg, David W Wetter

**Affiliations:** 1 Department of Biomedical Informatics University of Utah Salt Lake City, UT United States; 2 College of Nursing University of Utah Salt Lake City, UT United States; 3 Division of Epidemiology University of Utah Salt Lake City, UT United States; 4 Entertainment Arts and Engineering University of Utah Salt Lake City, UT United States; 5 Department of Psychiatry University of Utah Salt Lake City, UT United States; 6 Center for Health Outcomes and Population Equity Huntsman Cancer Institute Salt Lake City, UT United States

**Keywords:** diabetes prevention program, mobile video, motivation and problem solving, program enrollment, participant engagement, prediabetes

## Abstract

**Background:**

More than 88 million Americans are at risk of developing type 2 diabetes mellitus (T2DM). The National Diabetes Prevention Program’s Lifestyle Change Program (DPP LCP) has been shown to be effective in reducing the risk of progressing from prediabetes to T2DM. However, most individuals who could benefit from the program do not enroll.

**Objective:**

The aim of this trial is to test the real-world efficacy of 3 mobile phone–based approaches to increasing enrollment in the DPP LCP including a best-practice condition and 2 novel approaches.

**Methods:**

We will conduct a 3-armed randomized clinical trial comparing enrollment and 1-month engagement in the DPP LCP among adults with prediabetes from 2 health care settings. Participants in the best-practice condition will receive SMS-based notifications that they have prediabetes and a link to a website that explains prediabetes, T2DM, and the DPP LCP. This will be followed by a single question survey, “Would you like the DPP LCP to call you to enroll?” Participants in the 2 intervention arms will receive the same best-practice intervention plus either 2 mobile 360° videos or up to 5 brief phone calls from a health coach trained in a motivational coaching approach known as Motivation and Problem Solving (MAPS). We will collect measures of diabetes-related knowledge, beliefs in the controllability of risk for T2DM, risk perceptions for T2DM, and self-efficacy for lifestyle change pre-intervention and 4 weeks later. The primary outcomes of the study are enrollment in the DPP LCP and 4-week engagement in the DPP LCP. In addition, data on the person-hours needed to deliver the interventions as well as participant feedback about the interventions and their acceptability will be collected. Our primary hypotheses are that the 2 novel interventions will lead to higher enrollment and engagement in the DPP LCP than the best-practice intervention. Secondary hypotheses concern the mechanisms of action of the 2 intervention arms: (1) whether changes in risk perception are associated with program enrollment among participants in the mobile 360° video group and (2) whether changes in self-efficacy for lifestyle change are associated with program enrollment among participants in the MAPS coaching group. Finally, exploratory analyses will examine the cost effectiveness and acceptability of the interventions.

**Results:**

The project was funded in September 2020; enrollment began in February 2021 and is expected to continue through July 2022.

**Conclusions:**

We are conducting a test of 2 novel, scalable, mobile phone–based interventions to increase enrollment in the DPP LCP. If effective, they have tremendous potential to be scaled up to help prevent T2DM nationwide.

**Trial Registration:**

ClinicalTrials.gov NCT04746781; https://clinicaltrials.gov/ct2/show/NCT04746781

**International Registered Report Identifier (IRRID):**

DERR1-10.2196/28884

## Introduction

### Background

Among US adults, 34.5% have prediabetes, placing them at increased risk of type 2 diabetes mellitus (T2DM) [1]. Extensive evidence has shown that therapeutic lifestyle changes can reduce the progression from prediabetes to T2DM by 58% [2]. To address this national epidemic, the Centers for Disease Control and Prevention (CDC) has established the National Diabetes Prevention Program’s Lifestyle Change Program (DPP LCP) [3]. However, through 2019, only 0.4% of the 88 million adults in the United States with prediabetes have enrolled in the DPP LCP [4].

There are several reasons for low enrollment and engagement in the DPP LCP. First, many people with prediabetes are unaware of their risk for T2DM or do not believe that they are at risk of developing T2DM or its associated complications. Second, many individuals are not aware of the appropriate lifestyle changes that can prevent progression to T2DM [5-8]. This is important because risk perceptions are predictive of health behavior change [9]. Finally, several studies have identified practical barriers to enrolling in the DPP LCP, including the cost of enrollment, limited time for attendance, and difficulty with travel to and from DPP LCP sessions [10,11].

Most prior research on DPP LCP enrollment interventions has tested the effectiveness of medical providers notifying their patients of their prediabetes, counseling them, and referring them to the DPP LCP. These studies have reported DPP LCP enrollment rates of 8%-11% [12,13]. We believe there are significant limitations to this approach. First many providers do not notify their patients that they have prediabetes — only 15.3% of individuals with prediabetes report being told about their condition from a health professional [1]. Second, many providers do not currently counsel their patients about lifestyle changes, [14,15], likely because they do not feel they have the time or because they do not feel that such counseling will be effective [16,17].

In this project, we propose to address these issues by directly connecting with individuals with prediabetes through mobile phone–based interventions in a 3-armed randomized controlled trial. We will compare a best practice intervention with 2 novel interventions our research group has developed and pilot tested: mobile 360° videos and Motivation and Problem Solving (MAPS)–based phone counseling.

### Aims and Objectives

Aim 1 is to conduct a 3-armed randomized clinical trial comparing enrollment and 1-month engagement in the DPP LCP among adults with prediabetes receiving risk notification and education alone, risk notification and education plus the mobile 360° video, and risk notification and education plus MAPS.

Aim 1.1 is to examine the mechanisms underlying the mobile 360° video by comparing changes in deliberative, experiential, and affective risk perceptions across study arms.

Aim 1.2 is to examine the mechanisms underlying MAPS by comparing changes in DPP LCP–related self-efficacy across study arms.

## Methods

### General Methods

As noted in the previous section, whether an individual enrolls in the DPP LCP is a multifactorial process that includes their awareness, knowledge, risk perception, and unique barriers. Our intervention is designed to address each of the factors. First, to address low awareness of prediabetes, individuals with a diagnosis of prediabetes within the past 5 years documented in their electronic health record (EHR) will be informed via text message that they have prediabetes and sent a link to the website, Do I Have Prediabetes [18], that includes 3 components: a self-assessment of risk, didactic pages about prediabetes and T2DM, and didactic pages about the National DPP LCP and its benefits.

Individuals will then be randomized to receive only the initial best practice intervention, the best practice plus the mobile 360° videos, or the best practice plus MAPS counseling. The proposed mechanism of action for the mobile 360° videos divides risk perceptions into *deliberative* risk perceptions (ie, the individual's estimates of the likelihood of developing a condition), *affective* risk perceptions (ie, the individual's level of worry about a particular risk), and *experiential* risk perceptions (ie, how easy it is to imagine developing a condition). The videos are hypothesized to increase participants' affective (emotional) and experiential (gist-based) risk perceptions and the likelihood of enrolling in the DPP LCP. The proposed mechanisms of action for MAPS counseling include increasing both motivation and self-efficacy (eg, by addressing practical barriers to enrolling and engaging in the National DPP LCP).

The DPP LCP is an evidence-based program that is available at hundreds of locations throughout the United States as well as online [19]. It is a therapeutic lifestyle change program that reduces the risk of progression to T2DM by 58% for high-risk individuals [20]. The year-long lifestyle change program is comprised of weekly group meetings for 6 months followed by monthly meetings for another 6 months (a minimum total of 22 hours over the year) [1].

### Intervention

#### Best Practice Condition

In the best practice condition, we will send a text message to individuals who, according to their health records, have prediabetes. This message will notify them that they have prediabetes and refer them to a website that provides information about prediabetes and the effectiveness of the DPP LCP. This component of the intervention will increase participants’ awareness of the condition and the DPP LCP without adding to the workload of primary care clinicians. Additionally, this is a process that can be automated to regularly reach new individuals with prediabetes within a health system. While it is not yet standard clinical practice to notify individuals with prediabetes of their condition and offer the DPP LCP to them, there is evidence that individuals with prediabetes who are notified about their status are more likely to engage in self-directed healthy lifestyle changes [21]. Additionally, the American Diabetes Association 2018 Standards of Medical Care in Diabetes recommend that all patients with prediabetes “should be referred to an intensive behavioral lifestyle intervention program modeled on the Diabetes Prevention Program.” This recommendation has an evidence level of “A” [22].

#### Mobile 360° Videos

**Figure 1:**
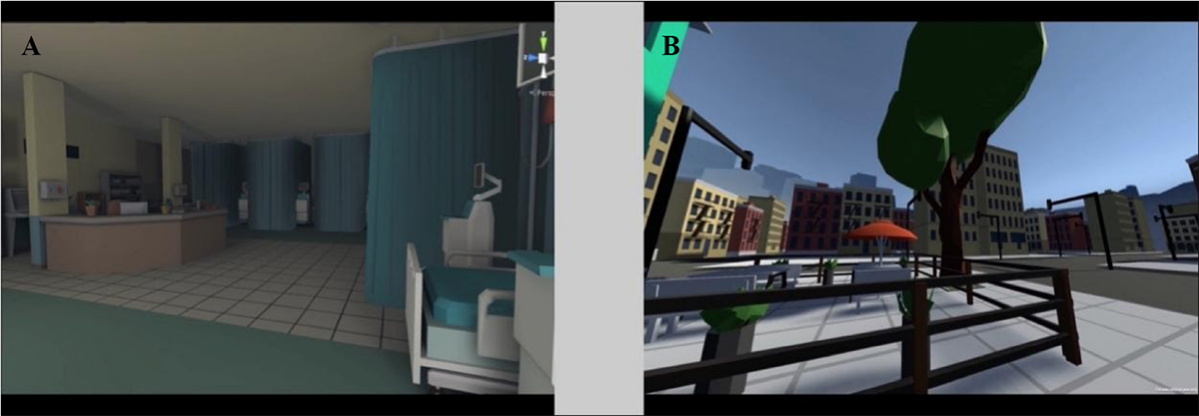
Screenshots of mobile 360° videos.

The mobile 360° videos (in which the viewer moves their phone to “look around” the world of the video) are intended to increase individuals’ risk perceptions regarding the potential adverse outcomes that might occur should they develop T2DM. Our team has designed and pilot tested two 3-minute videos with accompanying voiceover (in either English or Spanish) and soundtrack. In the first video (Figure 1A), the narrator describes the effect of diabetes on health and family life as an individual progresses from having prediabetes to T2DM and having a heart attack. The second video (Figure 1B) provides a vicarious experience of the changes in vision that occur as diabetic retinopathy progresses and uses the visual metaphor of building height to change risk perceptions: As the viewer is transported through a cityscape from a roof to progressively higher roofs, they develop visual scotomas that worsen as the height of the buildings (reflecting their average glucose level) increases. Each video ends with a positive message that enrolling in the DPP LCP can reduce the risk of this potential negative future.

#### MAPS

MAPS is a hybrid counseling technique that combines motivational interviewing and social cognitive, practical problem-solving to enhance motivation and self-efficacy for behavior change. MAPS has been shown to help people effectively participate in evidence-based programs and change their health behaviors [23,24]. Aside from our pilot work (manuscript in progress for the MAPS pilot and manuscript under review for the mobile 360° videos), these interventions had not been previously evaluated as tools to promote the DPP LCP.

A standardized MAPS training and treatment manual has been developed and used in pilot work and will be utilized in this project. Coach training will include both online “classroom” instruction and practice coaching sessions with feedback from a MAPS expert (KL).

The MAPS counseling itself will focus on promoting enrollment and engagement in the DPP LCP by addressing each participant’s unique values and barriers. MAPS uses a combination of motivational enhancement and social cognitive approaches based on motivational interviewing and practical problem-solving approaches. All participants will receive up to 5 telephone counseling calls lasting approximately 10 minutes during the 4 weeks following study enrollment. The timing of the MAPS counseling calls will be negotiated between the participant and health coach, as has been done for other MAPS trials. The MAPS counselor will also help each participant develop an individualized “wellness plan” that may not only include goals related to DPP LCP enrollment and engagement but also other potential stressors and concerns (eg, transportation, interpersonal issues, family problems, financial concerns). Thus, MAPS will assist individuals with various life stressors that may ultimately affect their DPP LCP engagement. MAPS counseling will be conducted via telephone in Spanish or English, and information from each session will be documented in a secure REDCap database.

All MAPS counseling sessions will be audio-recorded, and the recordings will be uploaded into the study database (in REDCap). Ongoing training and monitoring of recorded calls will ensure that the delivered MAPS follows the protocol precisely. A MAPS expert will review at least one recording per coach per week and provide detailed feedback and guidance as necessary to the health coach.

### Setting

Study participants will be recruited from 2 sites: University of Utah Health and the Midvale Community Building Community Clinic (CBC). University of Utah Health is the Mountain West’s only academic health care system and includes 5 hospitals, 12 community clinics, and several specialty centers. The Midvale CBC is an outpatient community health clinic providing medical, dental, physical therapy, and mental health services to low-income and uninsured families, with a predominantly Spanish-speaking clientele.

### Participants

Spanish- and English-speaking patients ages 18-89 years from University of Utah Health and the Midvale CBC will be invited to participate in the study.

#### Inclusion Criteria

Patients will be considered eligible for the proposed trial if (1) they are aged 18-89 years, (2) have a diagnosis of prediabetes within the past 5 years documented in the EHR (ICD-10 code R73.03), and (3) have an email or mailing address and a mobile telephone number on record with the health system.

#### Exclusion Criteria

Patients will be excluded from the study if they have any of the following diagnoses: T2DM (ICD-10-CM E11), type 1 diabetes mellitus (ICD-10-CM Diagnosis E10), diabetes mellitus due to underlying condition (ICD-10 E08), drug or chemical induced diabetes mellitus (ICD-10 E09); gestational diabetes (ICD-10 024.4), neonatal diabetes mellitus (ICD-10 P70.2), or post-pancreatectomy diabetes mellitus (ICD-10 E13). Individuals who are currently pregnant will be excluded from the trial. The rationale for this exclusion is that these women would be excluded from participating in the National DPP LCP. In addition, individuals who do not have an email address or mailing address or who do not own a smartphone will be excluded from the trial, simply because they could not complete the trial.

### Study Design

This is a 3-arm stratified randomized clinical trial comparing enrollment and 1-month engagement in the DPP LCP among adults with prediabetes receiving risk notification and education alone vs risk notification and education plus mobile 360° video vs risk notification and education plus MAPS. It should be noted that this study is not powered nor designed to compare the effectiveness of the mobile 360° videos vs MAPS nor does the design allow for testing of the additive value of mobile 360° videos plus MAPS.

### Procedures

#### Overview

**Figure 2:**
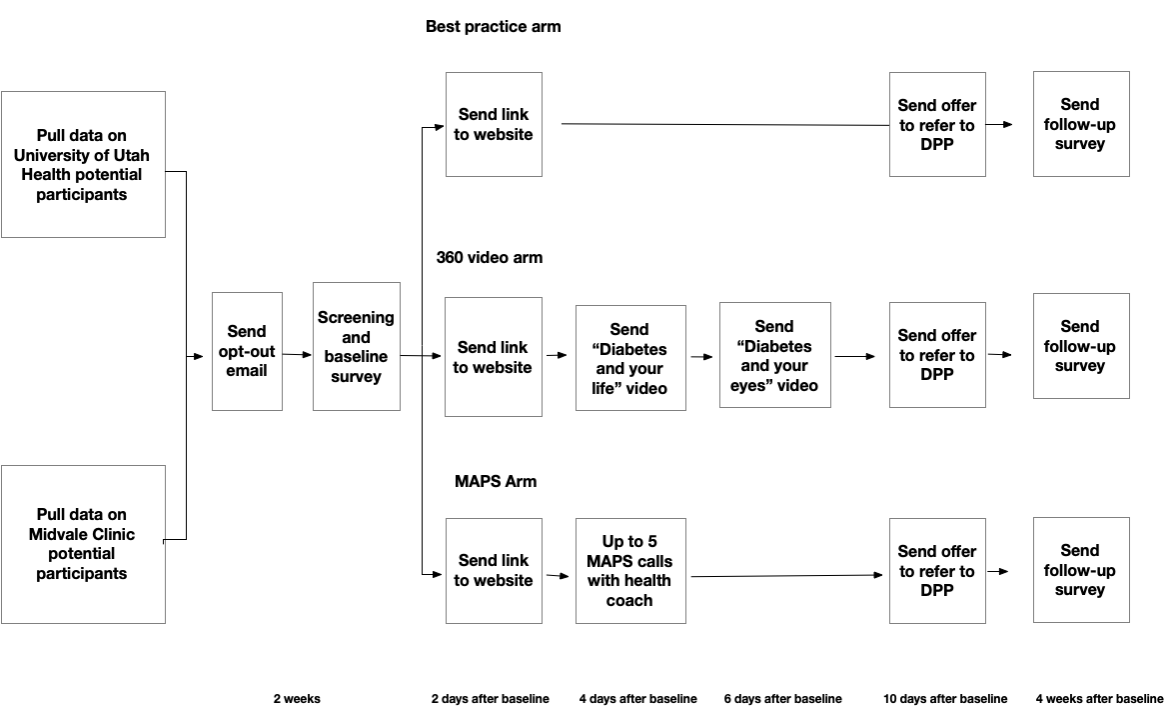
Overview of study procedures. DPP: Diabetes Prevention Program; MAPS: Motivation and Problem Solving.

Figure 2 provides an overview of the study procedures. We will begin by pulling lists of potential participants (using the inclusion and exclusion criteria listed in the previous sections) from the EHR of each health system. Selected individuals will be given the option to opt-out of any study-related communication prior to phone contact. Hence, we will first contact potential participants by email (or by letter if they do not have an email address on record) with a brief description of the study and an explanation that they will be contacted on their mobile phone in 2 weeks if they do not opt out. This email will include a link to click if they want to be excluded from further study communication. All further communication will be with individuals who did not opt out and will be sent via SMS messages to their mobile phone.

#### Screening and Baseline Survey

Potential participants who do not opt-out will then be sent an SMS message with a link to a survey. The survey will begin with a short eligibility screening section. Individuals who are screened out will be thanked for their time and informed that they do not meet the criteria for participating in the study.

Individuals who are not screened out will be directed to the consent cover letter and the remainder of the baseline survey. The baseline survey will collect information about demographics (age, sex, race/ethnicity, education, socioeconomic status), diabetes-related knowledge (11 items), and beliefs about the controllability of risk for type 2 diabetes (4 items) using 2 subscales on the risk perception for developing diabetes scale [25]. It will also collect information on self-efficacy related to DPP LCP participation as well as for diet, exercise, and weight loss (16 items) using the brief self-efficacy scales adapted from Wilson et al [26]. This will be followed by an 18-item scale of risk perceptions for T2DM that includes 6 items each to assess 3 different aspects of risk perception: deliberative, affective, and experiential risk perceptions [27].

#### Allocation to Condition

After completing the baseline survey, individuals will be allocated to the condition according to randomization tables generated by the projects statistician (YZ). These tables will be based on permuted block randomization, stratified by health system and sex, with block sizes randomly generated as 5, 10, or 15. In accordance with our planned sample sizes in each arm, each block will maintain a 1:2:2 ratio in sample size across the treatment groups (best practice, mobile 360° videos, and MAPS).

#### Risk Notification and Education

Two days after completing the baseline survey, all participants will be sent a hyperlink to the education website, Do I Have Prediabetes? [18]. This will be delivered as an iframe in a Qualtrics survey; in this way, we will be able to measure the number of participants who actually visit the website and the duration of time they spend on the site. After visiting the website, participants will be asked about which pages of the website they visited, what they learned, and what they liked and disliked about the website.

#### Mobile 360° Video

Four days after completing the baseline survey, participants randomized to the mobile 360° video arm will be sent the first video; 2 days later, they will be sent the second video. Up to 2 reminder texts 1 day apart will be sent to individuals who do not access the links for the videos. Similar to the educational website, the videos will be delivered as an iframe within a web-based survey. This will allow us to assess the duration of time spent watching the video and to collect immediate feedback on participants’ impressions of each video.

#### MAPS Phone Calls

A health coach trained in MAPS will call the participants randomized to that group; the first call may be used to initiate MAPS coaching or to simply schedule the upcoming MAPS counseling sessions. Participants will be offered up to 5 coaching calls over 4 weeks, each lasting approximately 10 minutes. During these sessions, participants will explore their motivation and goals and how enrollment in the DPP LCP may fit with these goals. Additionally, MAPS coaches will help participants identify barriers to achieving their goals and troubleshoot ways to address these barriers. If a participant enrolls in the DPP LCP, any remaining MAPS calls will serve to reinforce the positive change the individual has made, provide accountability, address ongoing challenges and barriers, and encourage continued engagement with the DPP LCP. All MAPS sessions will be audio-recorded, and at least 1 session per week per coach will be reviewed by a MAPS expert. Feedback will be provided to coaches as needed. This process will insure the fidelity of the MAPS intervention. Participants will have the option to tell the MAPS coaches that they would like to stop receiving calls at any point.

#### Offer to Refer to DPP LCP

Ten days after study enrollment, all participants will be sent a single question survey: “Would you like the DPP LCP to call you to help you enroll?” The contact information for participants who respond “yes” will be sent to the referral coordinator at their respective health system's DPP LCP, who will then call them to enroll. The rationale for this piece of the intervention is to mimic the current workflow for individuals to enroll — in most cases, a health care provider places a referral to the program via the EHR, and the program contacts the potential participant to enroll them. Due to the COVID-19 pandemic, both study sites have been offering the DPP LCP virtually, either as an asynchronous online program or through synchronous group sessions. During the study, participants will have the ability to select whatever DPP LCP format they prefer, including in-person sessions, if available.

#### Follow-Up Survey

The follow-up survey will be sent via text message to all participants 4 weeks after the baseline survey is completed. The survey will include all of the items described in the baseline survey (diabetes-related knowledge, beliefs about the controllability of diabetes, self-efficacy, and risk perceptions). The survey will conclude with a 9-item questionnaire about the practical barriers and facilitators that were relevant to choosing whether to enroll in the DPP LCP such as perceptions of the need for counseling to change one's lifestyle (which prior work suggests many individuals don't feel the need for) [28]; accessibility of the DPP LCP in terms of cost, location, time requirements, and scheduling [29]; and desire to participate in an online vs in-person DPP LCP [30,31]. This portion of the survey will provide space for free-text comments on each barrier and also ask for input on other barriers we did not anticipate. At the end of the follow-up survey, participants will be asked if they are willing to participate in a short phone-based semistructured interview about their experiences in the study. We will randomly select up to 40 individuals for these interviews; those who are interviewed will receive a US $20 electronic gift card.

We will use Qualtrics survey software for all questionnaires used in this study [32]. This platform allows us to automate the timing and delivery of each intervention component. This platform will allow us to assess the number of people contacted and the click rate of participants in each intervention component. Finally, the platform also provides a mechanism for participants to easily opt out of the trial at any time by responding “STOP” to any text message.

Each participant will be sent US $20 electronic gift cards upon completion of the baseline survey and follow-up survey (US $40 total for the study). To motivate completion of all study procedures, participants who complete both the baseline and follow-up questionnaires will be placed into a lottery for 1 of 5 US $100 electronic gift cards.

### Measures

#### Outcome Measures

The primary outcomes of the study are DPP LCP enrollment and 4-week engagement, as defined by the CDC (Table 1). These data are currently collected by all CDC-recognized organizations offering the National DPP LCP and are a part of their required reporting for DPP LCP recognition.

**Table 1:** Centers for Disease Control and Prevention (CDC)–defined measure of enrollment and engagement in in-person and online Diabetes Prevention Program’s Lifestyle Change Programs (DPP LCPs).

Time point	In-person	Online
Milestone 1 (enrollment)	Registration for the program	Setting their password for the app
Milestone 2 (4-week engagement)	Attending at least 2 out of the first 4 sessions	Must do at least 2 of the following activities: (1) Complete 2 education modules; (2) send at least 1 in-app message and/or Group Wall post; (3) set or log at least 1 behavior; (4) log, plan, or research at least 3 meals; (5) log physical activity at least 3 times; (6) weigh in on 3 or more days during 2 out of the first 4 sessions

The DPP LCP at University of Utah Health is covered by some insurance plans and requires an out-of-pocket fee for those without coverage. The cost for the online program is currently US $504, and the cost for the in-person program is US $425. Some scholarships are available for those who meet income guidelines. These costs may change during the course of the study. The Midvale CBC’s program is offered free of charge and is currently funded by a grant. To minimize the problem of cost as a barrier to enrollment, University of Utah Health participants who are uninsured or underinsured may be invited to participate in the Midvale CBC program. Otherwise, all other participants will only be invited to attend the program offered by their health system.

#### Process Measures

If the mobile 360° videos and MAPS are successful in increasing enrollment and early engagement with the DPP LCP as they have been in our pilot work (manuscript for the Video pilot study submitted to JMIR Diabetes, 11/26/20; manuscript for MAPS pilot in preparation), they have tremendous potential to be scaled up to help prevent T2DM nationwide. More than 80% of adults in the United States own a smartphone [33]. Therefore, health systems and DPP LCPs nationwide could implement these interventions. The scalability of the interventions depends, of course, not just on their efficacy but also on the effort that DPP LCPs must expend to implement them. Therefore, in this project, we will collect data on the person-hours required to implement each intervention at our 2 sites. This will inform our planned future work to test whether the intervention arms differ significantly in their effectiveness and cost effectiveness (Table 2).

**Table 2:** Process measures collected by study staff.

Measure	How measured
Number of participants who click on educational website link	Link is sent as a Qualtrics survey question; clicking on link results in data indicating a “click” was made.
Number of participants in the mobile 360° video group who click on each of the videos and time spent watching	Click rates from survey in which video is embedded and duration of time on each video
Number of participants in the MAPS group who respond to our initial attempts to connect for MAPS	Attempts to call will be documented by the health coach using a RedCAP survey.
Number of person-hours needed to train health coaches in MAPS	Documented hours for the duration of the training including practice sessions with KL
Number of person-hours required to deliver each MAPS session	The start and end time for each MAPS session will be documented by the health system using a REDCap survey.
Number of person-hours required to oversee MAPS sessions to insure fidelity to the protocol	Documented hours for the time spent by KL to review and provide feedback on a sample of recorded MAPS sessions

### Statistical Analyses

#### Sample Size and Power Calculation

Statistical power for the primary analysis in Aim 1 was calculated using the statistical power calculation software PASS 14 [34]. Based on prior work on the effect of risk notification and education, we hypothesize that 2% of participants in the risk notification and education arm of the study will enroll in the DPP LCP. In the intervention arms, based on our pilot work, we hypothesize that enrollment in the DPP LCP will increase to 32% and 45% for the mobile 360° video and MAPS arms, respectively (manuscript for the video pilot study submitted to JMIR Diabetes, 11/26/20; manuscript for MAPS pilot in preparation). We plan for a total sample size of 400; of whom, 80 will be randomized to risk notification and education alone, 160 will be randomized to risk notification and education plus mobile 360° video, and 160 will be randomized to risk notification and education plus MAPS. We calculated that this study will have >99% power to detect the hypothesized differences between the best practice arm and the intervention arms. To address the possibility of loss to follow-up, we are planning to recruit a total of 480 individuals into the trial.

#### Aim 1

We will use logistic regression to compare the effects of the 3 treatments on the likelihood of enrollment and 1-month engagement in the DPP LCP. The distributions of participants’ demographics and baseline measures of risk perception and self-efficacy will be summarized and compared by treatment groups using chi-squared tests for categorical variables and *F* tests for continuous variables. If participants’ demographics and baseline measures of risk perception are not balanced across the 3 treatment groups at baseline, we will use inverse probability of treatment weighting with propensity scores to create a synthetic sample and compare the difference in the causal average treatment effects across the 3 treatment groups [35]. We will calculate standardized differences to check whether the distribution of baseline covariates is independent of treatment groups conditional on the propensity score. We will conduct intention-to-treat analysis to account for noncompliance. We will summarize missing data for all variables in the dataset. We will employ multiple imputation to examine the impact of any missing values in a sensitivity analysis. All analyses will be conducted using statistical programming language R, and statistical significance will be defined at alpha=.05.

#### Aim 1.2

To examine the mechanisms underlying the mobile 360° video by comparing changes in deliberative, experiential, and affective risk perceptions across study arms, we will calculate the changes in risk perception scores across all 3 treatment groups using the scoring procedures described by Ferrer et al [27]. We will then use Baron and Kenny's [36] procedure to determine if the efficacy of the mobile 360° videos is mediated by changes in affective and experiential risk perceptions as hypothesized. The Baron and Kenny procedure assumes that the mediating factor is also randomly assigned to individuals in addition to the randomized baseline intervention (ie, sequential ignorability). However, the sequential ignorability assumption may not hold in this study even after adjusting for observed covariates. The potentially unmeasured confounders for the association between mediating factors and outcome may lead to biased inference. To reduce such bias, we will also employ 2 alternative casual modeling approaches, the structural mean model [37] and principal stratification [38,39], to evaluate how the impact of mobile 360° videos on DPP enrollment is mediated by risk perceptions.

#### Aim 1.3

To examine the mechanism of the MAPS intervention by comparing changes in DPP LCP–related self-efficacy across study arms**,** we will calculate the changes in self-efficacy across all 3 treatment groups using the scoring procedures described by Wilson et al [26]. We will then use a similar mediation procedure to the aforementioned to determine if the efficacy of MAPS is mediated by changes in self-efficacy for DPP LCP enrollment and engagement and self-efficacy for health behavior change as hypothesized [37]. Our hypothesis is that if MAPS increases participants’ self-efficacy, then enrollment in the DPP LPC will increase.

#### Process Measure Analysis

In addition to the efficacy data collected in the trial, the study team will collect a series of process measures (Table 2). We will summarize the distribution of these measures, conduct univariate analysis to explore the association between participants’ demographics and process measures, and calculate preliminary estimates of the cost of delivering each intervention component (person-time times the salary for that role) in order to prepare for future work that will compare the cost-effectiveness of our interventions at scale.

## Results

The project was funded in September 2020. The institutional review board approved the study on January 13, 2021. Enrollment began on March 10, 2021 and is expected to continue through July 31, 2022. As of April 27, 2021, 39 individuals had begun the study.

## Discussion

In this project, we will conduct a 3-arm randomized controlled trial intended to compare the efficacy of a best-practice approach and 2 novel interventions on enrollment and engagement in the DPP LCP. These interventions will be delivered directly to individuals with prediabetes through their mobile phones. To standardize the offer of referral to the DPP LCP, all participants will receive an offer to be referred to the National DPP LCP 10 days after completing the baseline survey. Our primary outcome will be participants' enrollment and 1-month engagement in the National DPP LCP.

### Strengths

This study is a randomized trial targeting patients with prediabetes within 2 health systems, including a community clinic serving primarily low-income, Spanish-speaking patients and a large academic medical center. The interventions will be delivered by mobile phones, making them scalable to large, diverse populations if found to be effective. The interventions will be delivered in English and Spanish, expanding the generalizability of study findings. The use of a theoretical framework for the study and validated questionnaires to elucidate the mechanism of action of the 2 novel interventions is a strength, as is the collection of process measures.

### Limitations

This study is not powered nor designed to compare the effectiveness of the mobile 360° video vs MAPS nor does the design allow for testing of the additive value of mobile 360° video plus MAPS. Because each of these interventions is quite novel, we felt that first they should have demonstrated incremental efficacy over the best-practice condition. If these interventions are found to be incrementally effective, future trials will be designed to compare the effectiveness of these interventions head-to-head as well as to evaluate a combination of the interventions (ie, via a sequential multiple assignment randomized trial [SMART] design). In addition, the differences in number of contacts between the best-practice condition, the video condition, and the MAPS condition may present a confound, making it unclear if outcomes differ due to number of contacts or intervention content. Finally, in this trial, we are only able to assess 1-month DPP LCP engagement. Future work should explore whether these interventions impact longer-term (12 months) engagement and health outcomes.

### Contingency Plans

Potential challenges we may encounter include difficulties with recruitment and retention. If we have difficulties with recruitment, we will expand our recruitment population to individuals with A1C levels between 5.7% and 6.4% rather than just those with a documented prediabetes ICD-9 code. If we have challenges with recruitment, we will revisit the frequency and timing of contact with participants. If many returned surveys have significant amounts of missing data, we will follow up with participants and ask them to respond to missing items. Additionally, if we are unable to meet our recruitment goal at the smaller Midvale CBC, we will enroll uninsured or underinsured Spanish-speaking patients from the University of Utah to supplement the Midvale sample.

### Future Work

All of the data will be used to inform the design of a large, SMART in which individuals will first receive risk notification and education; then, nonresponders will be randomized to mobile 360° video or nothing, and subsequent nonresponders will be randomized to MAPS phone counseling. Primary outcomes will be enrollment and 1-year engagement in the DPP LCP. Secondary outcomes will include changes in an objective measure of risk for T2DM including hemoglobin A_1c_ and weight. In this future trial, we will also compare the cost effectiveness of these interventions.

## Abbreviations

CBC: Community Building Community Clinic
CDC: Centers for Disease Control and Prevention
DPP LCP: Diabetes Prevention Program’s Lifestyle Change Program
EHR: electronic health record
MAPS: Motivation and Problem Solving
SMART: sequential multiple assignment randomized trial
T2DM: type 2 diabetes mellitus
